# *Cordyceps militaris* Treatment Preserves Renal Function in Type 2 Diabetic Nephropathy Mice

**DOI:** 10.1371/journal.pone.0166342

**Published:** 2016-11-10

**Authors:** Sung-Hsun Yu, Navneet Kumar Dubey, Wei-Shan Li, Ming-Che Liu, Han-Sun Chiang, Sy-Jye Leu, Ying-Hua Shieh, Feng-Chou Tsai, Win-Ping Deng

**Affiliations:** 1 Graduate Institute of Medical Sciences, College of Medicine, Taipei Medical University, Taipei, Taiwan; 2 Stem Cell Research Center, Taipei Medical University, Taipei, Taiwan; 3 Graduate Institute of Biomedical Materials and Tissue Engineering, College of Biomedical Engineering, Taipei Medical University, Taipei, Taiwan; 4 Department of Urology, Taipei Medical University Hospital, Taipei, Taiwan; 5 Department of Urology, School of Medicine, College of Medicine, Taipei Medical University, Taipei, Taiwan; 6 Division of Urology, Department of Surgery, Cathay General Hospital, New Taipei City, Taiwan; 7 Graduate Institute of Basic Medicine, Fu Jen Catholic University, New Taipei City, Taiwan; 8 Department of Microbiology and Immunology, School of Medicine, College of Medicine, Taipei Medical University, Taipei, Taiwan; 9 Department of Family Medicine, Taipei Medical University, Wan Fang Hospital, Taipei, Taiwan; 10 Cosmetic Clinic Group, Taipei, Taiwan; Hospital Universitario de la Princesa, SPAIN

## Abstract

Diabetic nephropathy is derived from long-term effects of high blood glucose on kidney function in type 2 diabetic patients. Several antidiabetic drugs and herbal medications have failed to prevent episodes of DN. Hence, this study aimed to further investigate the renal injury-reducing effect of antidiabetic CmNo1, a novel combination of powders of fruiting bodies and mycelia of *Cordyceps militaris*. After being administered with streptozotocin-nicotinamide and high-fat-diet, the diabetic nephropathy mouse model displayed elevated blood glucose and renal dysfunction markers including serum creatinine and kidney-to-body weight ratio. These elevated markers were significantly mitigated following 8 weeks CmNo1 treatment. Moreover, the chronic hyperglycemia-induced pathological alteration in renal tissue were also ameliorated. Besides, immunohistochemical study demonstrated a substantial reduction in elevated levels of carboxymethyl lysine, an advanced glycation end product. Elevated collagenous deposition in DN group was also attenuated through CmNo1 administration. Moreover, the enhanced levels of transforming growth factor-β1, a fibrosis-inducing protein in glomerulus were also markedly dampened. Furthermore, auxiliary risk factors in DN like serum triglycerides and cholesterol were found to be increased but were decreased by CmNo1 treatment. Conclusively, the results suggests that CmNo1 exhibit potent and efficacious renoprotective action against hyperglycemia-induced DN.

## Introduction

Diabetic nephropathy (DN), a diabetes-related insidious renal outcome represents the leading cause of end stage renal disease (ESRD) in adults affecting one-third population, thus causing an escalated economic burden on affected individual’s health-care [1]. According to a Reutens et al., the global prevalence of diabetes mellitus (DM) is estimated to increase from 6.4% (285 million) in 2010 to 7.7% (439 million) in 2030 out of which more than 90% will suffer from type 2 diabetes [2]. In a seminal report, approximately one third population of type 1 and type 2 diabetic patients have been documented to manifest the symptom of nephropathy in the form of impaired renal growth and development [3]. Hence, based on the above reports, an ever-increasing risk of nephropathy incidences in diabetic patients is estimated.

The DN is characterized by glomerular basement membrane (GBM) thickening, mesangial and tubulointerstitial expansion or hypertrophy [3, 4], scarring of renal glomerulus, known as glomerulosclerosis [5]. Mounting evidence indicates that under chronic hyperglycemic condition, the accumulated advanced glycation end products (AGEs) which are consequence of non-enzymatic reactions between sugar and free amino group of proteins, lipids and nucleic acids, promotes the generation of reactive oxygen species (ROS) leading to oxidative stress [6–9]. AGEs also stimulates the release of pro-sclerotic cytokines like transforming growth factor β1 (TGF-β1) [10], thereby participating in pathogenesis of renal injury, in particular, fibrosis [11].

Nowadays, the identification and development of the novel therapeutic alternatives to prevent the rapidly rising natural course of diabetic nephropathy are still underway. Of note, many antidiabetic drugs have failed to prevent treatment of DN [12] and may result in kidney failure as well [13]. In the recent years, concerted efforts have been made to explore the valuable biofactory of traditional Chinese herbal medicines as therapeutic alternatives owing to their lower side effects [14]. Interestingly, in addition to herbal medication, toxic renal outcome have also been reported [15]. However, *Cordyceps militaris* (CM), a *cordyceps* species accommodating various bioactive components is gaining much attention as it possess hypoglycemic [16], immunomodulating [17], antioxidant, antibacterial, antifungal and anti-tumor properties [18]. The majority of therapeutic properties of CM has been attributed to both, the fruiting bodies and mycelia [14, 19]. Furthermore, the fruiting body extract have been reported to possess greatest hypoglycemic activity than other strains and components but owing to host specificity and rarity in nature the fruiting bodies are much expensive [20, 21]. In our previous study, antidiabetic activity of CmNo1 has already been demonstrated [22]. So, in this report, we aimed to investigate whether CmNo1 also possess renoprotective effect.

To our knowledge, we herein provide the first experimental evidence on renoprotective activity of CmNo1, a novel combination of fruiting body and mycelia of CM, in high-fat diet and streptozotocin (STZ)–nicotinamide (NA)-induced type 2 diabetic mice. Specifically, the renal survival activities of CmNo1 on diabetic nephropathy were examined through histopathologic observations and renal function associated indexes including SCr and kidney weight to body weight ratio (KW/BW). The level of AGE and TGF-β1 were determined to corroborate oxidative stress and fibrotic activity respectively. Additionally, the other risk factors participating in progression of DN, for instance, triglyceride, and cholesterol levels were also evaluated.

## Materials and Methods

### Preparation of *Cordyceps militaris* No.1 (CmNo1) Extract

The commercially pulverized crude powders of the combined fruiting body and mycelium of *Cordyceps militaris* (CM), denoted as CmNo1 used in this study was provided by Liwanli Innovation Co., Ltd. (Taipei, Taiwan). CmNo1 contained 179.0 mg/g polysaccharides, 33.66 mg/g cordycepic acid, 0.91 mg/g adenosine, and 9.49 mg/g cordycepin. To determine the quantities of adenosine and codycepin present in CmNo1, the high-performance liquid chromatography (HPLC) DAD (Hitachi Co., Japan) analysis was performed while polysaccharide and cordycepic acid was quantified by UV-VIS spectroscopy. Thereafter, a suspension of CmNo1 was prepared with 2% carboxymethylcellulose (CMC; C4888, Sigma-Aldrich, USA) solution in water and administered orally to mice by stomach gavage at a dose of 360 mg/kg/day for 8 week.

### Animal Preparation and Experiment Design

C57BL/6J mice were purchased from National Laboratory Animal Center, Taipei, Taiwan and were maintained at Laboratory Animal Center, Taipei Medical University (TMU). All the animal care and use protocols were in accordance with guidelines of TMU Institutional Animal Care and Use Committee (IACUC) and prior approval was obtained by IACUC to conduct this study (approval no. LAC-2016-0011). After 1 week of acclimatization, the mice on high-fat diet (HFD), (58Y1, DIO Rodent Purified Diet, TestDiet) with 61.6% fat (3.140 Kcal) were daily injected intraperitoneally with 180 mg/kg nicotinamide (NA; N3376, Sigma-Aldrich, USA) 15 minutes prior to an intraperitoneal injection of 60 mg/kg streptozotocin (STZ; S0130, Sigma-Aldrich, USA) freshly dissolved in citrate buffer (pH 4.5) to induce substantial hyperglycemia for 4 weeks. Fasting blood glucose (FBG) values were checked weekly to confirm induction of diabetes in which blood samples were collected from tail-vein of normal control (NC) and STZ-NA administrated mice on HFD and was measured by glucose oxidase strips (Easytouch; Taiwan). The mice with blood glucose level exceeding 250 mg/dl were considered diabetic [23] and divided into two groups. One was used as diabetic nephropathy (DN) group (n = 6) while other group (n = 6) was treated with CmNo1, denoted as CmNo1-DN, at a dose of 360 mg/kg body weight/day via oral gavage. NC group were fed with normal chow (LabDiet 5010, 5.5% fat).

### Oral Glucose Tolerance Test (OGTT)

The OGTT was performed on weekly basis in the NC, DN, and CmNo1-DN mice during 8 consecutive weeks CmNo1 treatment using a standard method [24]. Mice were fasted overnight followed by oral administration of 30% solution (3g/kg) of D-glucose. Blood samples were collected from each group at 0, 30, 60, 90, 120 and 180 minutes relative to the start of the oral glucose administration for measuring blood glucose levels. The area under the glucose tolerance curve (Δ AUC_glucose_) was calculated using the trapezoidal rule to determine the integrated glucose response to the glucose load.

### Intraperitoneal Insulin Tolerance Test (IPITT)

Using blood samples obtained from tail veins of mice, glucose level was measured. Insulin (Humulin®, USA) was then injected intraperitoneally (0.75 IU/kg body weight) and blood glucose was measured again at intervals of 30, 60, 90, 120 and 180 min.

### Assessment of Kidney Dysfunction Markers: Serum Creatinine (SCr) and Kidney Hypertrophy Index

Following 8 weeks of CmNo1 treatment, whole blood was collected via retro-orbital sinus of each mouse into heparinized microhematocrit tubes (“Assistant”-Micro-Hematocrit-tubes 563, lot no. 1311444) and left undisturbed for 30 min at room temperature. The clot was removed through centrifugation at 1500 rpm for 15 min at room temperature. The serum was transferred into polypropylene tube and stored at -80°C. In order to assess the diabetic nephropathy, SCr levels were analyzed using Creatinine (serum) Colorimetric Assay Kit (700460, Cayman Chemical). The indices were examined spectrophotometrically. Further, the kidney hypertrophy index, another renal dysfunction marker was measured by kidney weight to body weight ratio [KW/BW (%)].

### Renal Histopathological Analysis

At the end of experiment, all the mice were anesthetized with an intravenous injection of tiletamine/zolazepam (Zoletil 30 mg/kg; Virbac, France) and xylazine (Rompun 10 mg/kg; Bayer HealthCare, Germany) and subjected to necropsy. Every possible effort was made to minimize animal suffering and lessen the number of animal use. Renal tissues were fixed in 10% formalin solution and embedded in paraffin for histopathological examination. The paraffin blocks were cut at 5 μm and later deparaffinized in xylene and rehydrated through graded alcohols. Thereafter, kidney slices of NC, DN and CmNo1-DN group were stained with hematoxylin & eosin (H&E) to examine cellular architecture, periodic acid schiff (PAS) for glycogen accumulation and masson’s trichrome (MT) to demonstrate the deposition of collagen in extracellular matrix (ECM). The tissue sections were then analyzed through light microscope. Image analysis of histologic sections were done using Digimizer and Image J softwares.

### Determination of Advanced Glycation End Products (AGEs) and Transforming Growth Factor-β1 (TGF-β1) in Renal Tissues

The renal levels of AGEs was determined by immunohistochemistry (IHC) assay, using monoclonal antibody reacting with N (epsilon)-(Carboxymethyl) lysine (CML) (1:500; Abcam 125145), a major AGE. Specifically, detection of signals in the paraffin-embedded tissues of mice kidney was performed with Vectastain ABC kit (Vector Laboratories) according to the manufacturer’s instructions as described. Renal tissue slices were used to perform immunohistochemical staining for TGF-β1 using rabbit polyclonal anti-TGF-β1 (1:500; GeneTex, Irvine, CA, USA).

### Western Blot Analysis

Following 8 week CmNo1 treatment, C57BL/6J mice from each group were sacrificed. For protein extraction, renal tissues were isolated and suspended in 100 μl 1X RIPA lysis buffer (Cat. No. 20–188, Millipore, USA), protease inhibitor cocktail set III, EDTA-Free (Cat. No. 539134, Millipore, USA), phosphatase inhibitor (Na_3_VO_4_), and sonicated. After 20 minutes incubation on ice, samples were centrifuged at 12000 rpm for 40 minutes at 4°C and supernatant was collected for quantification. The protein samples were resolved on 10% SDS-PAGE gel and transferred to a PVDF (Polyvinylidene Fluoride, Amersham Hybond-P, GE healthcare, UK) membrane. After blocking, membranes were incubated for 1 h at room temperature in PBST buffer with the TGF-β1 (1:500; GeneTex, Irvine, CA, USA) antibody. This was followed by 4 times wash for 10 minutes each at room temperature. Horseradish peroxidase-conjugated anti-rabbit IgG secondary antibody (1:10000; Jackson ImmunoResearch, West Grove, PA, USA) was diluted in PBST (PBS with 0.05% Tween-20, рН 7.0) and incubated with blots for 1 h at room temperature. Immunoreactivity expressions of TGF-β1 was measured by developing blots using ECL plus-kit (Amersham Pharmacia, USA). Blots were visualized by UVP BioSpectrum® imaging system while their densities were analyzed with VisionWorks LS software.

### Measurement of Metabolic Disturbances through Blood Biochemical Indices

Blood samples were collected via the retro-orbital sinus of mice before and after CmNo1 treatment. Samples were centrifuged and serum was collected from each of them. Serum levels of and triglyceride, high-density lipoprotein (HDL) and total cholesterol levels were measured by chemistry analyzer (FUJI DRI-CHEM 4000i). The low-density lipoprotein (LDL) was estimated by using Friedewald formula [LDL = (total cholesterol)-(HDL)-(triglyceride)/5] [25].

### Statistical Analysis

Data are represented as mean ± SD of for each group (NC, n = 5, DN, n = 6; CmNo1-DN, n = 6). The differences between 3 groups (NC, DN, CmNo1-DN) were estimated with two-way ANOVA (GraphPad Prism 6.0) and student’s t-test (SigmaPlot Version 10.0). Symbols specify significant difference from DN with *, ** and *** indicate *p*< 0.05, *p*< 0.01 and *p* < 0.001, respectively.

## Results

### Effect of CmNo1 on Glucose Level in Type 2 Diabetes Mellitus

**Fig 1:**
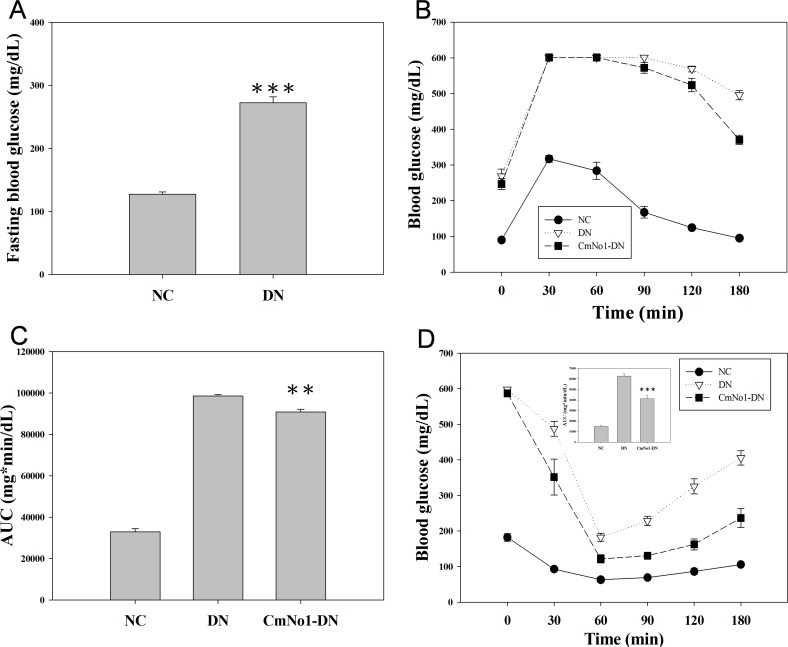
Effect of CmNo1 on glucose and insulin tolerance. (A) Fasting blood glucose (B) Assessment of glucose tolerance by oral glucose tolerance test (OGTT) from 0–180 min post-glucose loading during 8-week CmNo1 treatment (C) Total area under curves (AUC) for OGTT (D) Blood glucose values during intra-peritoneal insulin tolerance test over a period of 0–180 min during 8-week CmNo1 treatment. Data are expressed as mean ±SEM (NC, n = 5; DN, n = 6; CmNo1-DN, n = 6). Symbols specify significant difference from DN with ** and *** indicate *p*< 0.01 and *p* < 0.001, respectively. The solid circle and open inverted triangle denotes NC and DN respectively while solid square indicate CmNo1-DN mice. NC: Normal control; DN: Diabetic nephropathy; CmNo1-DN: Diabetic nephropathy group mice treated with *Cordyceps militaris* No1.

DN mouse model was established by administration of STZ/NA and high-fat-diet in NC mice. The diabetic status such as FBG levels were markedly raised to an average of 272.7±9.35 mg/dL in DN group (n = 12) compared to NC with a normoglycemic value of 127.4±3.53 mg/dL (n = 5) (Fig 1A). To evaluate the improvement in glucose tolerance ability, oral glucose tolerance test was performed. In 30 minutes after glucose administration, blood glucose reached the highest levels in all the groups and subsequently began to diminish; however, in 180 min, the CmNo1-DN group strongly recovered glucose tolerance compared to NC (Fig 1B). The result could be evidenced by AUC in which the CmNo1-DN group was significantly lower than DN (Fig 1C). We next accessed the peripheral insulin sensitivity through an intraperitoneal insulin tolerance test in which CmNo1-DN group showed a significant decrease in blood glucose level (Fig 1D), indicating that the treatment of CmNo1 could enhance glucose utilization and insulin sensitivity in DN mice.

### Effect of CmNo1 on Renal Function

**Fig 2:**
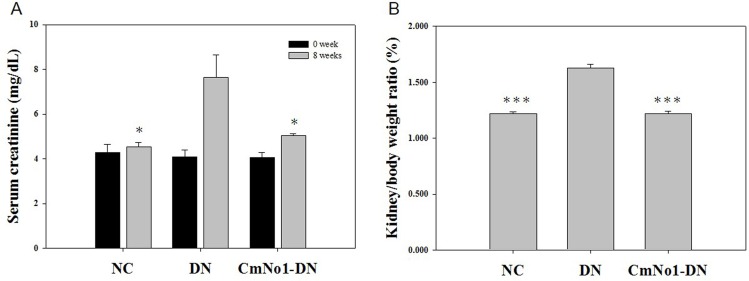
Efficacy of CmNo1 on kidney dysfunction markers. (A) Serum creatinine (B) Weight ratio of kidney to body (KW/BW). Data are expressed as mean ±SEM (NC, n = 5; DN, n = 6; CmNo1-DN, n = 6). Symbols specify significant difference from DN with * and *** indicate *p*< 0.05 and *p* < 0.001, respectively. NC: Normal control; DN: Diabetic nephropathy; CmNo1-DN: Diabetic nephropathy group mice treated with *Cordyceps militaris* No1.

Serum creatinine (SCr) is measured as standard indicator of renal function. In our study, DN group demonstrated a dramatically elevated SCr (7.63±1.01 mg/dL) compared to NC (SCr: 4.54±0.17 mg/dL), which indicated renal dysfunction. However, the administration of CmNo1 remarkably reduced SCr level 5.03±0.103 mg/dL (Fig 2A).

### The Weight Ratio of Kidney to Body

The kidney weight to body weight ratio (KW/BW), an important parameter for renal hypertrophy and dysfunction, was measured. We found a noticeable increase in KW/BW ratio of DN group compared to NC (Fig 2B). However, this increase was markedly attenuated to normal range following 8 weeks of CmNo1 intervention.

### Effects of CmNo1 on Hyperglycemia-Induced Renal Ultrastructure

**Fig 3:**
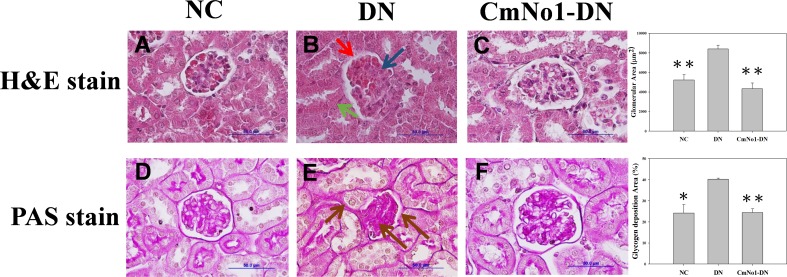
Effect of CmNo1 on ultrastructural alterations and glycogen accumulation. Representative microphotographs of kidney sections of NC, DN and CmNo1-DN group mice subjected to histologic H&E (A-C, ×1000) and PAS (D-F, ×1000) stain. NC: Normal control; DN: Diabetic nephropathy; CmNo1-DN: Diabetic nephropathy group mice treated with *Cordyceps militaris* No1; H&E, Hematoxylin and Eosin; PAS, Periodic Acid–Schiff.

To examine hyperglycemia-induced kidney injury, renal morphology was analyzed under light microscopy using hematoxylin and eosin (H&E) staining of renal tissue slices. The histological examination of H&E stained DN group (Fig 3A–3C) demonstrated swollen glomeruli, narrowed Bowman’s capsule, diffuse thickening of parietal layer of glomerular basement membrane with irregular distortions (red arrow), expanded mesangial regions (blue arrow) and indistinct peritubular capillaries (green arrow). In agreement with kidney dysfunction markers analysis, treatment with CmNo1 efficaciously re-established the normal morphology of glomeruli.

### Impact of CmNo1 on Renal Glycogen Accumulation

Membrane infolding, atrophy and apoptosis of renal tubules has been attributed to glycogen accumulation. The PAS staining (Fig 3D–3F) showed glomerulosclerosis in DN group due to intense glycogen deposition in mesangial regions, GBM and peritubular areas (brown arrow) which was also ameliorated in CmNo1-treated DN mice.

### Impact of CmNo1 on Renal Advanced Glycation End Product (AGE)

**Fig 4:**
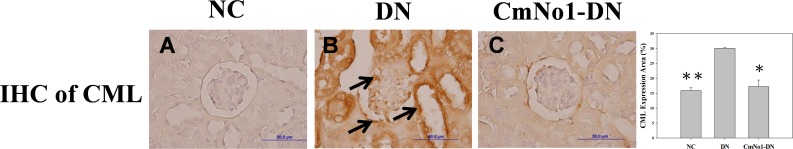
Efficacy of CmNo1 on carboxymethyl lysine (CML), an advanced glycation end product (AGE). immunohistochemial staining of CML of kidney sections of NC, DN and CmNo1-DN group (A-C, ×1000).

With immunohistochemical staining method, we further investigated whether the hyperglycemia-induced AGE were increased in kidney. As revealed through immunochemical staining (Fig 4A–4C), intense brown-color represented N^ϵ^-carboxymethyl lysine (CML), the AGE in glomeruli, GBM and tubular regions (black arrow) of DN group mice. Importantly, the CML staining was significantly minimized in kidneys of CmNo1-treated DN group indicating reduced accumulation of renal AGEs; while NC sections revealed no staining.

### Influence of CmNo1 on TGF-β1 Expression in Kidney Tissue

**Fig 5:**
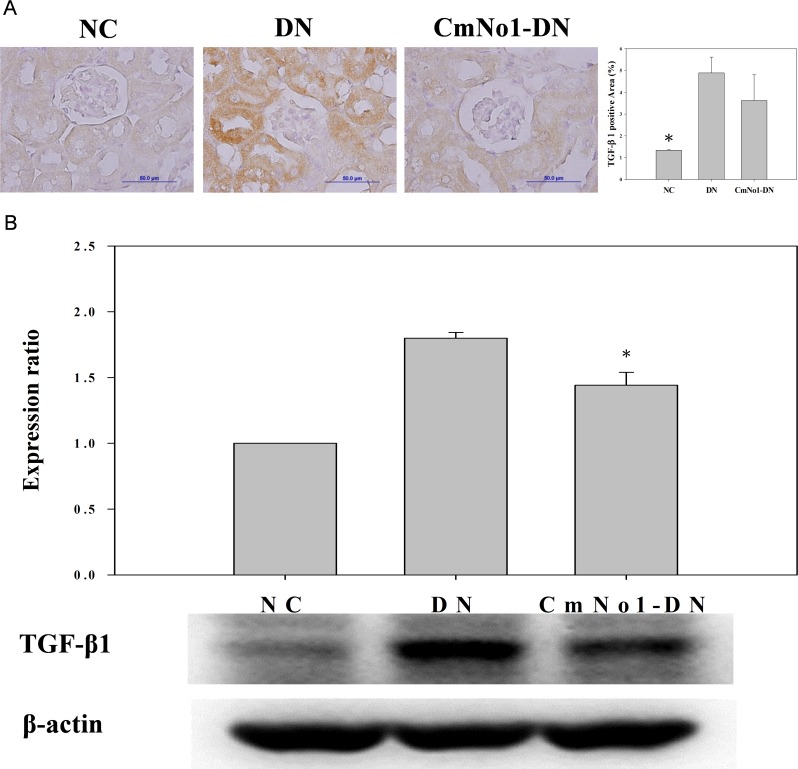
Immunohistochemical staining of TGF-β1 protein in mouse kidney. (A) TGF-β1 immunostaining in NC, DN and CmNo1-DN group mice while (B) Western blot profile of TGF-β1 protein expression and quantification. Symbols specify significant difference from DN with * indicate *p* < 0.05. NC: Normal control; DN: Diabetic nephropathy; CmNo1-DN: Diabetic nephropathy group mice treated with *Cordyceps militaris* No1.

The TGF-β1 participates in renal fibrosis, mesangial hypertrophy and accumulation of ECM. The results demonstrated a heavy deposition of TGF-β1 (brown color) in GBM and tubular compartments in DN group (Fig 5A, DN panel) which was reduced after CmNo1 administration. In agreement with immunohistochemical results, the immunoblotting of TGF-β1 revealed higher expression in the kidney tissues of DN group than the NC, which was effectively diminished by CmNo1 administration (Fig 5B, CmNo1-DN panel). The relative expression of TGF-β1 protein and quantification have also been shown in Fig 5B.

### Effects of CmNo1 on Induced Renal Collagenous Deposition

**Fig 6:**
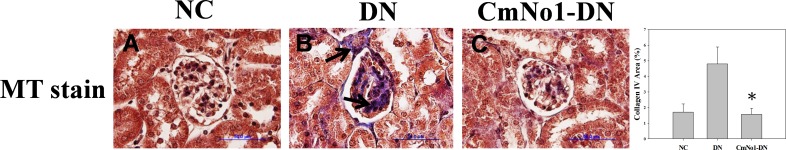
Effect of CmNo1 on collagenous deposition. Representative microphotographs of kidney sections of NC, DN and CmNo1-DN group mice subjected to MT (A-C, ×1000) stain. NC: Normal control; DN: Diabetic nephropathy; CmNo1-DN: Diabetic nephropathy group mice treated with *Cordyceps militaris* No1; MT, Masson trichrome.

In order to confirm glomerulosclerosis, kidney sections stained with MT (Fig 6A–6C) demonstrated elevated deposition of type IV collagen (gray to blue color) in the glomeruli, predominantly in mesangial regions (black arrow) as well as in tubular areas of DN group compared to NC, which was significantly alleviated in CmNo1-administered DN mice.

### Effect of CmNo1 on Serum Indices

**Fig 7:**
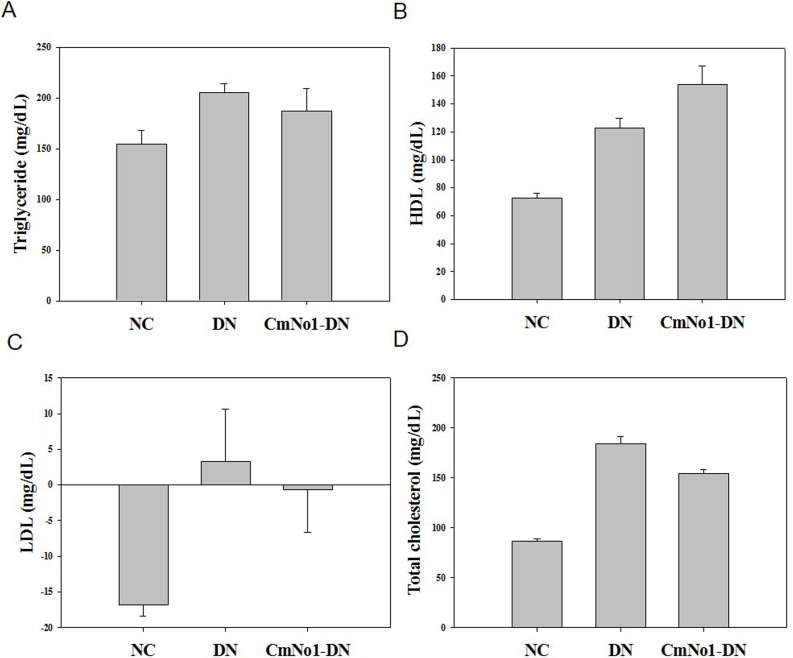
Effect of CmNo1 on metabolic disturbances after 8 week treatment. (A) Triglyceride (B) HDL (C) LDL (D) Total cholesterol. NC: Normal control; DN: Diabetic nephropathy; CmNo1-DN: Diabetic nephropathy group mice treated with *Cordyceps militaris* No1. HDL: High-density lipoprotein; LDL: low-density lipoprotein.

Serum insulin, triglyceride, and cholesterol levels have also been reported to be other characteristic features of renal impairments in hyperglycemic conditions [26, 27]. Our results showed the triglyceride, high-density lipoprotein (HDL), low-density lipoprotein (LDL) and total cholesterol levels in the DN group were found to be 205.25±8.86 mg/dL, 123.60±6.81 mg/dL, 3.16±5.67 mg/dL and 184.5±6.51 mg/dL respectively, which were significantly higher than those of their respective NC group (triglyceride: 154.33±14.06 mg/dL; HDL: 77.83±3.07 mg/dL, LDL: -16.86±1.58 mg/dL and total cholesterol: 86.83±1.90 mg/dL). Following CmNo1 administration, a considerable reduction in levels of triglyceride, LDL, total cholesterol but elevation in HDL were found (triglyceride: 187.66±21.76 mg/dL; HDL: 153.75±13.28 mg/dL, LDL: -0.6±6.086 mg/dL and total cholesterol: 154.66±3.48 mg/dL) (Fig 7A, 7B, 7C & 7D).

## Discussion

Kidneys play a role in removal of toxic products from the body and maintain fluid, minerals and electrolyte balance at physiological level. But an elevated blood glucose level could be a prognostic factor for damage to the various segments of the nephron causing chronic kidney disorders [28]. Other prior studies also strongly support that hyperglycemia poses higher risk of nephropathy [29, 30]. However, various antidiabetic drugs and herbal administration have proven inefficacy in preventing DN and leads to further complications [12, 15]. Hence, the current study extended to elucidate renoprotective activity of antidiabetic CmNo1 (already described in previous report) [22]. The mouse model exhibited metabolic abnormalities (hypoinsulinemia, hypertriglyceridemia, and hypercholesterolemia) and renal lesions resembling DN in humans [31]. Since this model imitated pathophysiology of DN in humans; we hypothesized whether CmNo1 could exert renoprotective effect.

The major pharmacological agent in CmNo1 preparations was determined with high content of polysaccharide (191.79 mg/g). In a very important study, the polysaccharide fraction of CM has also been reported to exhibit higher hypoglycemic activity than *O*. *sinensis* and other species [16]. In another study, anti-fibrotic action have also been attributed to polysaccharides [32]. In a report by Zhang et al., polysaccharide reduced the activation of the transcription factor, nuclear factor-κB (NF-κB), which is attributed to be a key step in the pathogenesis of DN [33]. Hence, it indicates the polysaccharide fraction present in the CmNo1 exerted hypoglycemic effect possibly via the activation of a key regulator of insulin action through peroxisome proliferator-activated receptor gamma (PPAR-γ) [34] and NF-κB. The additional chemical components such as cordycepin (3'-deoxyadenosine) and adenosine were also present at higher levels which were 9.49 mg/g and 0.6 mg/g respectively. Besides, other reports have demonstrated cordycepin induced anti-fungal [35], anti-malarial [36] and hypoglycemic activities [37]. In a seminal study, cordycepin (7.24 mg/g) have been demonstrated to exert immunomodulatory and tumor delaying effect [38]. Another active constituent, cordycepic acid have shown prophylactic action against postoperative acute renal failure, cough and asthma, and anti-free radical activities [39]. Besides, the adenosine present in *cordyceps* have been documented to inhibit reactive oxygen species (ROS) thereby reducing oxidative stress [40]. Based on these previous reports on therapeutic activities of the bioactive constituents contained in CmNo1, we conclude that CmNo1 exerts beneficial effect for the treatment of diabetic nephropathy. In our study, administration of CmNo1 for consecutive 8 weeks significantly inhibited the elevated blood glucose (Fig 1B, 1C and 1D). Owing to economical and little inconvenience, SCr, a most widely used and reliable clinical marker of compromised renal function [41, 42] was also reduced (Fig 2A).

During progression of diabetes, escalated kidney weight to body weight (KW/BW) is another harbinger of renal hypertrophy [43] showing kidney injury and renal insufficiency. Our result demonstrated that compared to NC, KW/BW ratio in DN group was significantly increased which was ameliorated in CmNo1-treated DN group suggesting substantial anti-hypertrophic effect (Fig 2B).

Besides, in DN, abnormalities in glomerular and tubular structures are among the most striking and consistently identified alterations. Our findings of light microscopy demonstrated that compared to normal kidney structure of NC group, DN group kidneys (Fig 3B) clearly demonstrated extreme mesangial expansion, segmental glomerulosclerosis and diffuse thickening of GBM, leading to facilitate the downward spiral of pathological symptoms. Additionally, renal tubular membrane infolding, atrophy and their apoptosis renal tubules has been attributed to glycogen accumulation [44]. However, supplementation with CmNo1 for 8 weeks significantly recovered altered morphology (Fig 3C) and further reduced accumulated glycogen in kidneys of DN group (Fig 3F) which indicated rescuing effect on renal structure and function.

Moreover, elevated synthesis of advanced glycation end products (AGEs) in hyperglycemic environment have been reported to cause irreversible damage to kidney tissues through overproduction of reactive oxygen species (ROS) via oxidative stress [6, 45]. Further, it has been noted that the carboxymethyl lysine (CML), one of the AGE structures generated under oxidative condition is frequently measured in processed foods and living bodies as a marker of glycation and oxidation [46, 47]. Specifically, CML is generated by the oxidative cleavage of Amadori products by hydroxyl radicals [48] and peroxynitrite [49] which suggests that CML is an indispensable biological marker of oxidative stress in vivo. In kidney sections of DN group, we detected an intense accumulation of carboxymethyl lysine (CML) as a glycated product (Fig 4B) which was significantly attenuated in CmNo1-treated DN group (Fig 4C). This result signals anti-oxidative activity through CmNo1-mediated renoprotection which is in agreement with a previous study demonstrating efficacy of cordycepin exhibiting anti-oxidation [50]. This is also supported by a previous study describing the role of polysaccharide in scavenging free-radicals produced during oxidative stress [51].

A host of mediators like hyperglycemia, glycated proteins, growth factors and cytokines have been reported to participate in cascade of pathologic events ending in DN [52]. Of these, patients with type 2 diabetes have been reported with higher expression of TGF-β1, a key profibrotic cytokine which accelerates the progression of renal fibrosis [53], glomerular mesangial hypertrophy [54], excessive amassing of extracellular matrix (ECM) [55]. Of note, in DN, TGF-β1 has been reported to induce abnormal collagenous deposition in extracellular matrix, a marker of glomerular sclerosis and interstitial fibrosis resulting into lessened surface area for glomerular filtration [56, 57]. Our immunochemical study and western blot analysis showed that CmNo1 greatly reduced TGF-β1 (Fig 5) and concomitant collagenous deposits (Fig 6C) in renal tissues. Since the role of collagenous deposition in GBM, glomerular mesangium and tubular interstitium indicating a biomarker of fibrotic activity has already been reported [58, 59], our results demonstrated ameliorated accumulation of mesangial matrix and collagen deposition which signified improved and quite normal appearance.

Besides, several experimental evidence have shown that hyperlipidemia is an independent risk factor for the progression of DN [60]. Enhanced triglycerides, serum LDL, total cholesterol, and reduced HDL have also been described as characteristic alterations in diabetic patients posing a major risk factor for cardiovascular events [26, 61–63]. Our results demonstrated that CmNo1 significantly attenuated triglycerides, LDL and TC while escalated the HDL levels in DN mice suggesting their anti-atherosclerotic action (Fig 7) [50]. Taken together, the result suggested an accelerated preventive and curative effects of CmNo1 against DN symptoms. However, our study has few limitations. Despite CmNo1 exhibited remedial and preventive intervention, further studies are needed to investigate the therapeutic efficacy of each component of CmNo1 to provide a deeper insight into their activity. Furthermore, though TGF-β1 was detected through IHC and western blotting, their urinary and serum concentration was not determined. In conclusion, CmNo1 possess a strong potential for treatment of type 2 diabetic nephropathy.

## References

[1] Cade WT. Diabetes-related microvascular and macrovascular diseases in the physical therapy setting. Physical therapy. 2008;88(11):1322–35. 10.2522/ptj.20080008 18801863 PMC2579903

[2] Reutens AT, Atkins RC. Epidemiology of diabetic nephropathy. Contributions to nephrology. 2011;170:1–7. 10.1159/000324934 . 21659752

[3] Schena FP, Gesualdo L. Pathogenetic mechanisms of diabetic nephropathy. Journal of the American Society of Nephrology: JASN. 2005;16 Suppl 1:S30–3. . 15938030 10.1681/asn.2004110970

[4] Fioretto P, Mauer M. Histopathology of diabetic nephropathy. Seminars in nephrology. 2007;27(2):195–207. 10.1016/j.semnephrol.2007.01.012 17418688 PMC2746982

[5] Qian Y, Feldman E, Pennathur S, Kretzler M, Brosius FC 3rd. From fibrosis to sclerosis: mechanisms of glomerulosclerosis in diabetic nephropathy. Diabetes. 2008;57(6):1439–45. 10.2337/db08-0061 18511444 PMC4239998

[6] Tan AL, Forbes JM, Cooper ME. AGE, RAGE, and ROS in diabetic nephropathy. Seminars in nephrology. 2007;27(2):130–43. 10.1016/j.semnephrol.2007.01.006 . 17418682

[7] Yu T, Robotham JL, Yoon Y. Increased production of reactive oxygen species in hyperglycemic conditions requires dynamic change of mitochondrial morphology. Proceedings of the National Academy of Sciences of the United States of America. 2006;103(8):2653–8. 10.1073/pnas.0511154103 16477035 PMC1413838

[8] Ha H, Hwang IA, Park JH, Lee HB. Role of reactive oxygen species in the pathogenesis of diabetic nephropathy. Diabetes research and clinical practice. 2008;82 Suppl 1:S42–5. 10.1016/j.diabres.2008.09.017 . 18845352

[9] Williamson JR, Chang K, Frangos M, Hasan KS, Ido Y, Kawamura T, et al Hyperglycemic pseudohypoxia and diabetic complications. Diabetes. 1993;42(6):801–13. . 8495803 10.2337/diab.42.6.801

[10] Yamagishi S, Matsui T. Advanced glycation end products, oxidative stress and diabetic nephropathy. Oxidative medicine and cellular longevity. 2010;3(2):101–8. 10.4161/oxim.3.2.11148 20716934 PMC2952094

[11] Deshpande SD, Putta S, Wang M, Lai JY, Bitzer M, Nelson RG, et al Transforming growth factor-beta-induced cross talk between p53 and a microRNA in the pathogenesis of diabetic nephropathy. Diabetes. 2013;62(9):3151–62. 10.2337/db13-0305 23649518 PMC3749352

[12] Norlin J, Nielsen Fink L, Helding Kvist P, Douglas Galsgaard E, Coppieters K. Abatacept Treatment Does Not Preserve Renal Function in the Streptozocin-Induced Model of Diabetic Nephropathy. PloS one. 2016;11(4):e0152315 10.1371/journal.pone.0152315 . 27055155 PMC4824484

[13] Nasri H, Rafieian-Kopaei M. Diabetes mellitus and renal failure: Prevention and management. Journal of research in medical sciences: the official journal of Isfahan University of Medical Sciences. 2015;20(11):1112–20. 10.4103/1735-1995.172845 26941817 PMC4755100

[14] Dong Y, Jing T, Meng Q, Liu C, Hu S, Ma Y, et al Studies on the antidiabetic activities of *Cordyceps militaris* extract in diet-streptozotocin-induced diabetic Sprague-Dawley rats. BioMed research international. 2014;2014:160980 10.1155/2014/160980 24738047 PMC3967809

[15] Akpan EE, Ekrikpo UE. Acute Renal Failure Induced by Chinese Herbal Medication in Nigeria. Case reports in medicine. 2015;2015:150204 10.1155/2015/150204 26199625 PMC4496464

[16] Zhang G, Huang Y, Bian Y, Wong JH, Ng TB, Wang H. Hypoglycemic activity of the fungi *Cordyceps militaris*, *Cordyceps sinensis*, *Tricholoma mongolicum*, and *Omphalia lapidescens* in streptozotocin-induced diabetic rats. Applied microbiology and biotechnology. 2006;72(6):1152–6. 10.1007/s00253-006-0411-9 . 16575562

[17] Hsu CH, Sun HL, Sheu JN, Ku MS, Hu CM, Chan Y, et al Effects of the immunomodulatory agent *Cordyceps militaris* on airway inflammation in a mouse asthma model. Pediatrics and neonatology. 2008;49(5):171–8. 10.1016/S1875-9572(09)60004-8 . 19133568

[18] Reis FS, Barros L, Calhelha RC, Ciric A, van Griensven LJ, Sokovic M, et al The methanolic extract of *Cordyceps militaris* (L.) Link fruiting body shows antioxidant, antibacterial, antifungal and antihuman tumor cell lines properties. Food and chemical toxicology: an international journal published for the British Industrial Biological Research Association. 2013;62:91–8. 10.1016/j.fct.2013.08.033 . 23994083

[19] Lo HC, Tu ST, Lin KC, Lin SC. The anti-hyperglycemic activity of the fruiting body of *Cordyceps* in diabetic rats induced by nicotinamide and streptozotocin. Life sciences. 2004;74(23):2897–908. 10.1016/j.lfs.2003.11.003 . 15050427

[20] Hong IP, Kang PD, Kim KY, Nam SH, Lee MY, Choi YS, et al Fruit Body Formation on Silkworm by *Cordyceps militaris*. Mycobiology. 2010;38(2):128–32. 10.4489/MYCO.2010.38.2.128 23956640 PMC3741563

[21] Hur H. Chemical Ingredients of *Cordyceps militaris*. Mycobiology. 2008;36(4):233–5. 10.4489/MYCO.2008.36.4.233 23997632 PMC3755201

[22] Ang L, Yuguang L, Liying W, Shuying Z, Liting X, Shumin W. Ergosterol Alleviates Kidney Injury in Streptozotocin-Induced Diabetic Mice. Evidence-based complementary and alternative medicine: eCAM. 2015;2015:691594 10.1155/2015/691594 26664454 PMC4664816

[23] Clee SM, Attie AD. The genetic landscape of type 2 diabetes in mice. Endocrine reviews. 2007;28(1):48–83. 10.1210/er.2006-0035 . 17018838

[24] Wang HY, Kan WC, Cheng TJ, Yu SH, Chang LH, Chuu JJ. Differential anti-diabetic effects and mechanism of action of charantin-rich extract of Taiwanese *Momordica charantia* between type 1 and type 2 diabetic mice. Food and chemical toxicology: an international journal published for the British Industrial Biological Research Association. 2014;69:347–56. 10.1016/j.fct.2014.04.008 . 24751968

[25] Huey-Jen Hsu S, Chen MF, Chen DR, Su TC. Validation of the Estimation of Low-density Lipoprotein Cholesterol by the Modified Friedewald Equation in Ethnic Chinese Adults Living in Taiwan. Internal medicine. 2015;54(18):2291–7. 10.2169/internalmedicine.54.4308 . 26370851

[26] Saito T. Abnormal lipid metabolism and renal disorders. The Tohoku journal of experimental medicine. 1997;181(3):321–37. . 9163848 10.1620/tjem.181.321

[27] Rave K, Heise T, Pfutzner A, Heinemann L, Sawicki PT. Impact of diabetic nephropathy on pharmacodynamic and Pharmacokinetic properties of insulin in type 1 diabetic patients. Diabetes care. 2001;24(5):886–90. . 11347749 10.2337/diacare.24.5.886

[28] Agarwal P. Management of diabetic kidney disease: Recent advances. Indian journal of endocrinology and metabolism. 2013;17(Suppl 1):S55–8. 10.4103/2230-8210.119506 24251221 PMC3830368

[29] Larkins RG, Dunlop ME. The link between hyperglycaemia and diabetic nephropathy. Diabetologia. 1992;35(6):499–504. . 1612221 10.1007/BF00400475

[30] Alaveras AE, Thomas SM, Sagriotis A, Viberti GC. Promoters of progression of diabetic nephropathy: the relative roles of blood glucose and blood pressure control. Nephrology, dialysis, transplantation: official publication of the European Dialysis and Transplant Association—European Renal Association. 1997;12 Suppl 2:71–4. . 9269705

[31] Yokozawa T, Nakagawa T, Wakaki K, Koizumi F. Animal model of diabetic nephropathy. Experimental and toxicologic pathology: official journal of the Gesellschaft fur Toxikologische Pathologie. 2001;53(5):359–63. 10.1078/0940-2993-00203 . 11817105

[32] Peng J, Li X, Feng Q, Chen L, Xu L, Hu Y. Anti-fibrotic effect of *Cordyceps sinensis* polysaccharide: Inhibiting HSC activation, TGF-beta1/Smad signalling, MMPs and TIMPs. Experimental biology and medicine. 2013;238(6):668–77. 10.1177/1535370213480741 . 23918878

[33] Zhang YW, Wu CY, Cheng JT. Merit of Astragalus polysaccharide in the improvement of early diabetic nephropathy with an effect on mRNA expressions of NF-kappaB and IkappaB in renal cortex of streptozotoxin-induced diabetic rats. Journal of ethnopharmacology. 2007;114(3):387–92. 10.1016/j.jep.2007.08.024 . 17900838

[34] Cho EJ, Hwang HJ, Kim SW, Oh JY, Baek YM, Choi JW, et al Hypoglycemic effects of exopolysaccharides produced by mycelial cultures of two different mushrooms *Tremella fuciformis* and *Phellinus baumii* in ob/ob mice. Applied microbiology and biotechnology. 2007;75(6):1257–65. 10.1007/s00253-007-0972-2 . 17457544

[35] Sugar AM, McCaffrey RP. Antifungal activity of 3'-deoxyadenosine (cordycepin). Antimicrobial agents and chemotherapy. 1998;42(6):1424–7. 9624488 10.1128/aac.42.6.1424 PMC105616

[36] Trigg PI, Gutteridge WE, Williamson J. The effects of cordycepin on malaria parasites. Transactions of the Royal Society of Tropical Medicine and Hygiene. 1971;65(4):514–20. . 4999656 10.1016/0035-9203(71)90162-3

[37] Yun Y, Han S, Lee S, Ko SK, Lee CK, Ha NJ, et al Anti-diabetic Effects of CCCA, CMESS, and Cordycepin from *Cordyceps militaris* and the Immune Responses in Streptozoitocin-induced Diabetic mice. Natural Products Sciences. 2003;9(4):291–8. Epub 2003. 12. 31.

[38] Jeong MH, Lee CM, Lee SW, Seo SY, Seo MJ, Kang BW, et al Cordycepin-enriched *Cordyceps militaris* induces immunomodulation and tumor growth delay in mouse-derived breast cancer. Oncology reports. 2013;30(4):1996–2002. 10.3892/or.2013.2660 . 23921598

[39] Lin S, Liu ZQ, Xue YP, Baker PJ, Wu H, Xu F, et al Biosynthetic Pathway Analysis for Improving the Cordycepin and Cordycepic Acid Production in *Hirsutella sinensis*. Applied biochemistry and biotechnology. 2016;179(4):633–49. 10.1007/s12010-016-2020-0 . 26922724

[40] Olatunji OJ, Feng Y, Olatunji OO, Tang J, Ouyang Z, Su Z, et al Neuroprotective effects of adenosine isolated from *Cordyceps cicadae* against oxidative and ER stress damages induced by glutamate in PC12 cells. Environmental toxicology and pharmacology. 2016;44:53–61. 10.1016/j.etap.2016.02.009 . 27114365

[41] Batlle D. Clinical and cellular markers of diabetic nephropathy. Kidney international. 2003;63(6):2319–30. 10.1046/j.1523-1755.2003.00053.x . 12753325

[42] Hosten AO. BUN and Creatinine 1990. Available: http://www.ncbi.nlm.nih.gov/books/NBK305/. 21250147

[43] Raz I, Wexler I, Weiss O, Flyvbjerg A, Segev Y, Rauchwerger A, et al Role of insulin and the IGF system in renal hypertrophy in diabetic Psammomys obesus (sand rat). Nephrology, dialysis, transplantation: official publication of the European Dialysis and Transplant Association—European Renal Association. 2003;18(7):1293–8. . 12808164 10.1093/ndt/gfg170

[44] Kang J, Dai XS, Yu TB, Wen B, Yang ZW. Glycogen accumulation in renal tubules, a key morphological change in the diabetic rat kidney. Acta diabetologica. 2005;42(2):110–6. 10.1007/s00592-005-0188-9 . 15944846

[45] Berg TJ, Bangstad HJ, Torjesen PA, Osterby R, Bucala R, Hanssen KF. Advanced glycation end products in serum predict changes in the kidney morphology of patients with insulin-dependent diabetes mellitus. Metabolism: clinical and experimental. 1997;46(6):661–5. . 9186302 10.1016/s0026-0495(97)90010-x

[46] Nagai R, Ikeda K, Higashi T, Sano H, Jinnouchi Y, Araki T, et al Hydroxyl radical mediates N epsilon-(carboxymethyl)lysine formation from Amadori product. Biochemical and biophysical research communications. 1997;234(1):167–72. . 9168983 10.1006/bbrc.1997.6608

[47] Uribarri J, Woodruff S, Goodman S, Cai W, Chen X, Pyzik R, et al Advanced glycation end products in foods and a practical guide to their reduction in the diet. Journal of the American Dietetic Association. 2010;110(6):911–16 e12. 10.1016/j.jada.2010.03.018 20497781 PMC3704564

[48] Nagai R, Nagai M, Shimasaki S, Baynes JW, Fujiwara Y. Citric acid inhibits development of cataracts, proteinuria and ketosis in streptozotocin (type 1) diabetic rats. Biochemical and biophysical research communications. 2010;393(1):118–22. 10.1016/j.bbrc.2010.01.095 20117096 PMC2917331

[49] Nagai R, Unno Y, Hayashi MC, Masuda S, Hayase F, Kinae N, et al Peroxynitrite induces formation of N (epsilon) -(carboxymethyl) lysine by the cleavage of Amadori product and generation of glucosone and glyoxal from glucose: novel pathways for protein modification by peroxynitrite. Diabetes. 2002;51(9):2833–9. . 12196478 10.2337/diabetes.51.9.2833

[50] Won KJ, Lee SC, Lee CK, Lee HM, Lee SH, Fang Z, et al Cordycepin attenuates neointimal formation by inhibiting reactive oxygen species-mediated responses in vascular smooth muscle cells in rats. Journal of pharmacological sciences. 2009;109(3):403–12. . 19305122 10.1254/jphs.08308fp

[51] Liu Y, Wang J, Wang W, Zhang H, Zhang X, Han C. The Chemical Constituents and Pharmacological Actions of *Cordyceps sinensis*. Evidence-based complementary and alternative medicine: eCAM. 2015;2015:575063 10.1155/2015/575063 25960753 PMC4415478

[52] Bundschuh I, Jackle-Meyer I, Luneberg E, Bentzel C, Petzoldt R, Stolte H. Glycation of serum albumin and its role in renal protein excretion and the development of diabetic nephropathy. European journal of clinical chemistry and clinical biochemistry: journal of the Forum of European Clinical Chemistry Societies. 1992;30(10):651–6. . 1493158

[53] Yanagita M. Inhibitors/antagonists of TGF-beta system in kidney fibrosis. Nephrology, dialysis, transplantation: official publication of the European Dialysis and Transplant Association—European Renal Association. 2012;27(10):3686–91. 10.1093/ndt/gfs381 23114895

[54] Jung Tak Park MK, Lanting Linda, Putta Sumanth, Castro Nancy and Natarajan Rama. TGF-beta activates Akt kinase and induces glomerular mesangial hypertrophy related to diabetic nephropathy through FOG2 inhibition by microRNA-200b/c FASEB J. 2012;26(759.3).

[55] Awazu M, Omori S, Ishikura K, Hida M, Fujita H. The lack of cyclin kinase inhibitor p27(Kip1) ameliorates progression of diabetic nephropathy. Journal of the American Society of Nephrology: JASN. 2003;14(3):699–708. . 12595506 10.1097/01.asn.0000051726.41601.c0

[56] Yokozawa T, Yamabe N, Cho EJ, Nakagawa T, Oowada S. A study on the effects to diabetic nephropathy of Hachimi-jio-gan in rats. Nephron Experimental nephrology. 2004;97(2):e38–48. 10.1159/000078405 . 15218322

[57] Lee HS. Pathogenic Role of TGF-β in Diabetic Nephropathy. Journal of Diabetes and Metabolism. 2013;S9:7 10.4172/2155-6156.S9-008

[58] Genovese F, Manresa AA, Leeming DJ, Karsdal MA, Boor P. The extracellular matrix in the kidney: a source of novel non-invasive biomarkers of kidney fibrosis? Fibrogenesis & tissue repair. 2014;7(1):4 10.1186/1755-1536-7-4 24678881 PMC3986639

[59] Choi YE, Ahn SK, Lee WT, Lee JE, Park SH, Yoon BB, et al Soybeans ameliolate diabetic nephropathy in rats. Evidence-based complementary and alternative medicine: eCAM. 2010;7(4):433–40. 10.1093/ecam/nen021 18955330 PMC2892345

[60] Rosario RF, Prabhakar S. Lipids and diabetic nephropathy. Current diabetes reports. 2006;6(6):455–62. . 17118229 10.1007/s11892-006-0079-7

[61] Pang J, Chan DC, Watts GF. Origin and therapy for hypertriglyceridaemia in type 2 diabetes. World journal of diabetes. 2014;5(2):165–75. 10.4239/wjd.v5.i2.165 24748930 PMC3990315

[62] Almquist T, Jacobson SH, Mobarrez F, Nasman P, Hjemdahl P. Lipid-lowering treatment and inflammatory mediators in diabetes and chronic kidney disease. European journal of clinical investigation. 2014;44(3):276–84. 10.1111/eci.12230 . 24720535

[63] Mulec H, Johnsen SA, Wiklund O, Bjorck S. Cholesterol: a renal risk factor in diabetic nephropathy? American journal of kidney diseases: the official journal of the National Kidney Foundation. 1993;22(1):196–201. . 8322783 10.1016/s0272-6386(12)70186-5

